# Programmed cell death protein-1 (PD-1) protects liver damage by suppressing IFN-γ expression in T cells in infants and neonatal mice

**DOI:** 10.1186/s12887-021-02794-x

**Published:** 2021-07-16

**Authors:** Xuangjie Guo, Yiping Xu, Wei Luo, Rongli Fang, Li Cai, Ping Wang, Yuxia Zhang, Zhe Wen, Yanhui Xu

**Affiliations:** 1 Department of Pediatric Surgery, Guangdong Provincial Key Laboratory of Research in Structural Birth Defect Disease, Guangdong Provincial Children’s Medical Research Center, Guangzhou Women and Children’s Medical Center, Guangzhou Medical University, Guangzhou, 510623 Guangdong China; 2 The Second Institute of Clinical Medicine, Guangzhou University of Chinese Medicine, Guangzhou, 510405 China

**Keywords:** Biliary atresia, PD-1, IFN-γ

## Abstract

**Background:**

Biliary atresia (BA) is a severe cholangiopathy possibly resulting from virus-induced and immune-mediated injury of the biliary system. IFN-γ, secreted from CD4^+^ Th1 cells and CD8^+^ cytotoxic T cells, is a major mediator of liver pathology. Programmed death protein-1 (PD-1) signaling suppresses T cell function. However, how PD-1 modify T cell function in BA remains incompletely understood.

**Methods:**

Frequencies of PD-1 expressing CD4^+^ and CD8^+^ T cells were analyzed in the liver and blood from BA and control subjects. Associations of PD-1^+^CD4^+^/CD8^+^T cell abundances with liver function indices were measured. Function of PD-1 was measured by administration of an anti-PD-1 antibody in a Rhesus Rotavirus (RRV)-induced BA model. Survival, histology, direct bilirubin, liver immune cell subsets and cytokine production were analyzed.

**Results:**

PD-1 was significantly upregulated in CD4^+^ and CD8^+^ T cells in patients with BA compared with control subjects. PD-1 expression in T cells was negatively associated with IFN-γ concentration in liver (PD-1^+^CD4^+^T cells in liver vs. IFN-γ concentration, *r* = − 0.25, *p* = 0.05; PD-1^+^CD8^+^T cells in liver vs. IFN-γ concentration, *r* = − 0.39, *p* = 0.004). Blockade of PD-1 increased IFN-γ expression in CD4^+^ T and CD8^+^ T cells (RRV vs. anti-PD-1 treated RRV mice: 11.59 ± 3.43% vs. 21.26 ± 5.32% IFN-γ^+^ in hepatic CD4^+^T cells, *p* = 0.0003; 9.33 ± 4.03% vs. 22.55 ± 7.47% IFN-γ^+^ in hepatic CD8^+^T cells, *p* = 0.0001), suppressed bilirubin production (RRV vs. anti-PD-1 treated RRV mice: 285.4 ± 47.93 vs. 229.8 ± 45.86 μmol/L total bilirubin, *p* = 0.01) and exacerbated liver immunopathology.

**Conclusions:**

PD-1 plays a protective role in infants with BA by suppressing IFN-γ production in T cells. Increasing PD-1 signaling may serve as a therapeutic strategy for BA.

**Supplementary Information:**

The online version contains supplementary material available at 10.1186/s12887-021-02794-x.

## Background

Biliary atresia (BA) is a disease of the biliary system presenting in infancy with poor prognosis and high mortality [1, 2]. If uncorrected, BA results in death within first 2 years of life. Although the symptoms could be partially improved by Kasai surgery, ~ 50% patients continue develop into liver failure and BA remains the major cause for childhood liver transplantation [3, 4]. A current hypothesis of BA pathogenesis is that a primary perinatal cholangiovirus infection initiates an autoimmune response against the bile duct epithelium, while a secondary chronic inflammation mediates bile duct injury and fibrosis [5]. Studies using patients’ biopsies and rotavirus-induced BA mouse models provide strong evidence for virus-induced autoimmune pathways [6, 7]. We [8] and others [9, 10] have shown that IFN-γ, secreted from CD4^+^ T helper 1 (Th1) cells, ex-CD4^+^ T helper 17 (Th17) T cells and CD8^+^ T cells, are involved in the pathogenesis of BA. IFN-γ^+^ cells are present within the portal tracts in the livers of infant with biliary atresia. Blockade of IFN-γ is sufficient to prevent liver damage in rhesus rotavirus (RRV)-induced BA models in neonatal mice [11]. These findings support the notion that Th1 cell–mediated inflammation plays an important role in BA pathogenesis.

Programmed cell death protein 1 (PD-1) is a critical inhibitory molecule that suppresses IFN-γ production by T cells and maintains peripheral tolerance [12]. When PD-1 or PD-L1 was deficient in NOD mice, onset and prevalence of type 1 diabetes (T1D) were accelerated, associating with an enhanced Th1 infiltration into the islets of pancreas [13]. In mouse model of autoimmune encephalomyelitis, blocking PD-1 or PD-L1/2 increased disease severity by promoting lymphocytes entry to the central nervous system [14]. Therefore, PD-1/PD-L1 appear to be involved in the development of autoimmune diseases in murine models. However, the role of PD-1 signaling in BA remains to be clarified.

Here, we examined PD-1 expression in patients with BA and in RRV-induced BA mice. We found that PD-1 was highly elevated in T cells in BA and its blockade augmented IFN-γ production and liver pathology.

## Materials and methods

### BA cohort

We recruited a cohort of children from the Department of Pediatric Surgery at Guangzhou Women and Children’s Medical Center between January 2020 and January 2021. BA was diagnosed according to intraoperative cholangiography when the intrahepatic biliary tree and/or extrahepatic bile duct could not be observed. All BA patients involved were full-term infants. Two groups of patients were included for controls: children with normal liver function but requiring surgery for choledochal cyst (CC) and age matched infants with neonatal hepatis syndrome (NHS).

### Flow cytometry

Liver biopsies obtained from patients during laparoscopy or Kasai’s operation were processed according to published procedures by Wang. et al. [9]. Peripheral blood mononuclear cells or hepatic lymphocytes were incubated for 45 min at 4 °C with saturating concentrations of fluorescently labeled antibodies and analyzed on a FACSAria SORP flow cytometer (BD Science). Lymphocyte gating was performed based on the forward and side scatter pattern, followed by dead cell exclusion using propidium iodide (PI) in all surface staining samples.

### RRV-induced mouse model of BA and anti-PD-1 treatment

All experimental wild-type BALB/c mice were SPF rated and placed in a room with a 12-h dark/light cycle. Mice were injected intraperitoneally (i.p.) at 12–18 h of birth with 30 μl (1.5 × 10^^6^ PFU/ml) rhesus rotavirus (RRV) or 0.9% saline (Control mice). RRV-infected mice that died within the first 2 days were excluded from the study. The clinical symptoms of BA development include yellowing of the skin and sclera, acholic stool, and growth retardation. RRV-infected mice were randomized into BA-RRV group or anti-PD-1 group. A total of four doses of anti-PD-1 antibody (50 mg/kg) were given by i.p. injection every 3 days, starting on day 1 of life. Mice were sacrificed at 12 days after RRV, saline, RRV + anti-PD-1 treatment.

### Liver function indices

Blood was collected by heart puncture and centrifuged at 13,000 r.p.m. 4 °C for 5 min. Serum was collected for liver function measurement. ALT, AST, DB, TB were measured with liver function Assay (an automated clinical chemistry analyzer (Beckman Coulter AU5811, USA)).

### Histopathology

The fresh liver tissues were fixed with 4% paraformaldehyde and embedded in paraffin, then sectioned (2 mm in thickness) and stained with hematoxylin and eosin (H&E) for morphological evaluation and Masson’s trichrome staining for fibrosis.

### Intracellular cytokine staining

Lymphocytes isolated from liver biopsies of patients or mice models were cultured on V-bottom 96-well plates in RPMI 1640 (Gibco) medium (R10) containing 10% heat-inactivated FBS (Gibco), 100 U/ml penicillin/streptomycin (Gibco) and 100 μM nonessential amino acids (Gibco) at 37 °C in 5% CO_2_. 100 ng/ml Phorbol 12-myristate 13-acetate (PMA, Sigma-Aldrich, St Louis, MO), 2 μM/ml Ionomycin (Sigma-Aldrich) and 2 μM/ml monensin (Sigma-Aldrich) were used to stimulate the lymphocytes for 4–6 h [9]. Intracellular staining was performed using eBioscience™ Intracellular Fixation & Permeabilization Buffer Set (Invitrogen) following the manufacturer’s protocol. Briefly, stimulated cells were surface stained prior to fixation on ice for 20–30 min. Then cells were stained for intracellular cytokines at 4°Cfor 1 h and resuspended in FACS buffer (PBS + 2%FBS) before analysis by flow cytometry.

### Statistical analysis

Prism 7.0 (GraphPad Software) was used for statistical analysis. Unpaired t test was used for two group analysis and one-way ANOVA for three or more group analysis. Non-parametric data were analyzed by Mann-Whitney U test or Kruskal-Wallis test. For all analyses, 2-tailed *p* values were calculated. *P* values for multiple comparisons were adjusted by the Benjamini- Hochberg method (Benjamini and Hochberg, 1995). All data points are shown with central lines indicating medians unless stated otherwise.

## Results

### PD-1 is highly expressed in T cells in the blood and liver of infants with BA

Table 1 displays characteristics of BA cohort and control groups. The population for this study consisted of 68 BA patients, 26 CC patients and 7 neonatal hepatis syndrome (NHS) who were recruited from the Department of Pediatric Surgery at Guangzhou Women and Children’s Medical Center between January 2020 and January 2021. The age (days) variables derived from the date of birth for an individual at the date of surgery. To get rid of its impact on immune phenotypes, we included age-matched NHS patients and divided CC patients into two groups: under 4-month-old (*n* = 9) as age-matched controls for BA, and older than 4-month-old (*n* = 17). Except for GGT, liver enzymes test showed no significant differences between BA and NHS patients included in this study. GGT, the most sensitive liver enzyme test for detecting bile duct injury, was significantly increased in BA patients. Patients with CC demonstrated normal liver function.

**Table 1:** Demographics of the BA and control patients enrolled in this study

Variables	BA (*n* = 68)	CC (≤4 months, *n* = 9)	CC (> 4 months, *n* = 17)	NHS (*n* = 7)
Age at surgery, mean (SD), days	70.62 ± 21.5	100 ± 25.98	1134 ± 553.3	58.29 ± 14.16
Sex, M/F	26/42	3/6	4/13	6/1
ALT, mean (SD), U/L	206.1 ± 143.7	66.11 ± 68.78^***^	58.38 ± 77.74^****^	211.2 ± 108.6^n.s^
AST, mean (SD), U/L	292.6 ± 183.3	88.67 ± 95.78^****^	59.00 ± 61.68^****^	223.4 ± 114.0^n.s^
GGT, mean (SD), U/L	658.5 ± 541.3	188.3 ± 294.5^***^	150.7 ± 241.5^****^	293.2 ± 331.3^*^
DBIL, mean (SD), μmol/L	175.2 ± 278.8	28.26 ± 50.41^****^	20.45 ± 28.67^****^	127 ± 35.17^n.s^
TBIL, mean (SD), μmol/L	163 ± 43.29	40.09 ± 69.41^****^	24.27 ± 34.19^****^	162.5 ± 54.19^n.s^

*BA* biliary atresia, *CC* choledochal cyst, *NHS* neonatal hepatis syndrome, *SD* standard deviation. Significance was determined by Mann-Whitney U test. n.s: not significant. ****p < 0.0001,***p < 0.001,**p < 0.01, *p < 0.05.

**Fig. 1:**
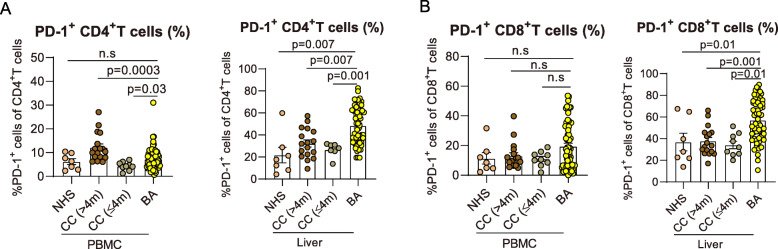
PD-1 expressing CD4^+^ and CD8^+^ T cells are increased in BA. **A** Dot plots showing frequencies of PD-1^+^CD4^+^ T cells in BA (*n* = 68) and control subjects (CC: > 4-month-old *n* = 17, ≤4-month-old *n* = 9; NHS: *n* = 7) . Horizontal lines indicate median values. Significance was determined by Mann-Whitney U test. **B** Dot plots showing frequencies of PD-1^+^CD8^+^ T cells in BA and control subjects. Horizontal lines indicate median values. Significance was determined by Mann-Whitney U test

To reveal relevance of PD-1 expression and disease process of BA, we first examined PD-1 expression on CD4^+^ T and CD8^+^ T cells from peripheral blood mononuclear cells (PBMC) and liver biopsies. Representative gating strategies were shown in Figure S1. We found that the percentages of PD-1 were significantly increased in hepatic CD4^+^ T cells (Fig. 1A) as well as blood and hepatic CD8^+^ T cells in infants with BA compared with control subjects (Fig. 1B).

### PD-1 expression is negatively associated with IFN-γ concentration in the liver of infants with BA

**Fig. 2:**
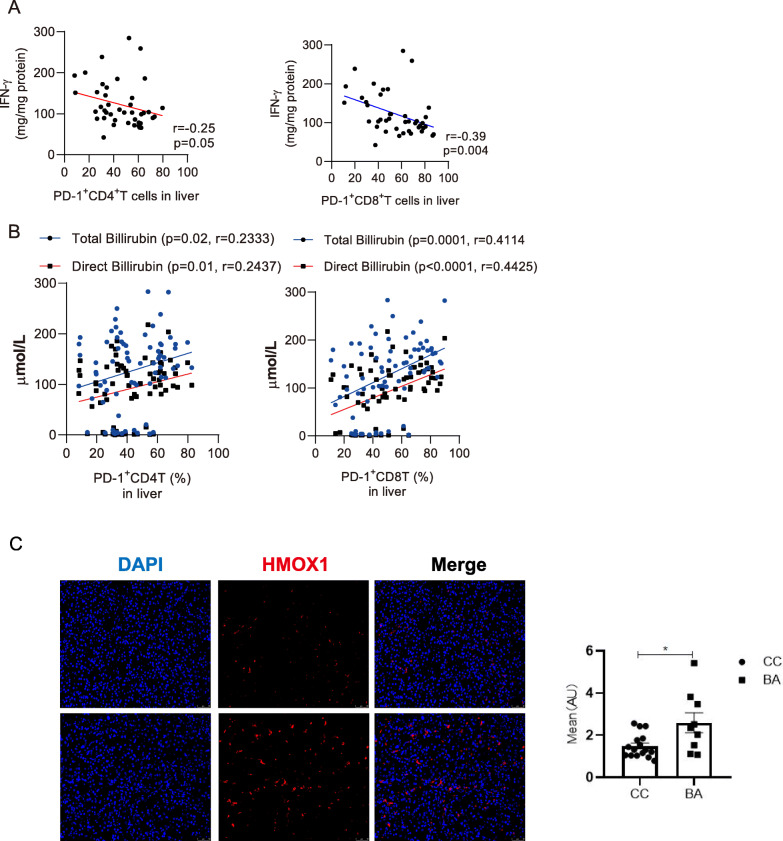
PD-1 expression is negatively associated with IFN-γ concentration in the livers of infants with BA. **A** Scatter plots showing correlation between concentrations of IFN-γ with the frequencies of PD-1^+^CD4^+^T and PD-1^+^CD8^+^T cells in livers from BA and CC infants. **B** Scatter plots showing correlation between concentrations of total bilirubin and direct bilirubin with the frequencies of PD-1^+^CD4^+^T and PD-1^+^CD8^+^T cells in livers from BA and CC infants. **C** Right panel: representative immunofluorescent images showing distribution of HMOX1 (red) in the liver from BA (*n* = 3) and CC (*n* = 3) subjects. Left panel: dot plots showing mean fluorescence intensity in the liver biopsies from BA compared with CC

To demonstrate the function of PD-1, we measured its expression with liver IFN-γ concentrations. We found that IFN-γ concentration in liver homogenates was negatively correlated with the frequencies of PD-1^+^CD4^+^ T and CD8^+^ T cells (Fig. 2A). Of liver function indices, we found that PD-1 expression was positively associated serum bilirubin concentrations (Fig. 2B). Furthermore, Heme oxygenase-1 (HMOX1), the rate-limiting enzyme that catalyzes generation of biliverdin (converted to bilirubin) [15], was found to be increased in liver of BA (Fig. 2C). Mildly increase of bilirubin has been shown as a potent anti-oxidant [16] and immune-suppressive agent of T cell activation [17]. Thus, we speculate that PD-1 upregulation may suppress IFN-γ expression in T cells and reduce oxidative damage via bilirubin. In addition, percentages of PD-1 expressing T cells were not associated with other liver enzymatic indices (ALT, AST, GGT, AKP) or time of native liver survival (Figure S2A-E).

### Blockade of PD-1 aggravates liver pathology in RRV-induced model of biliary atresia

**Fig. 3:**
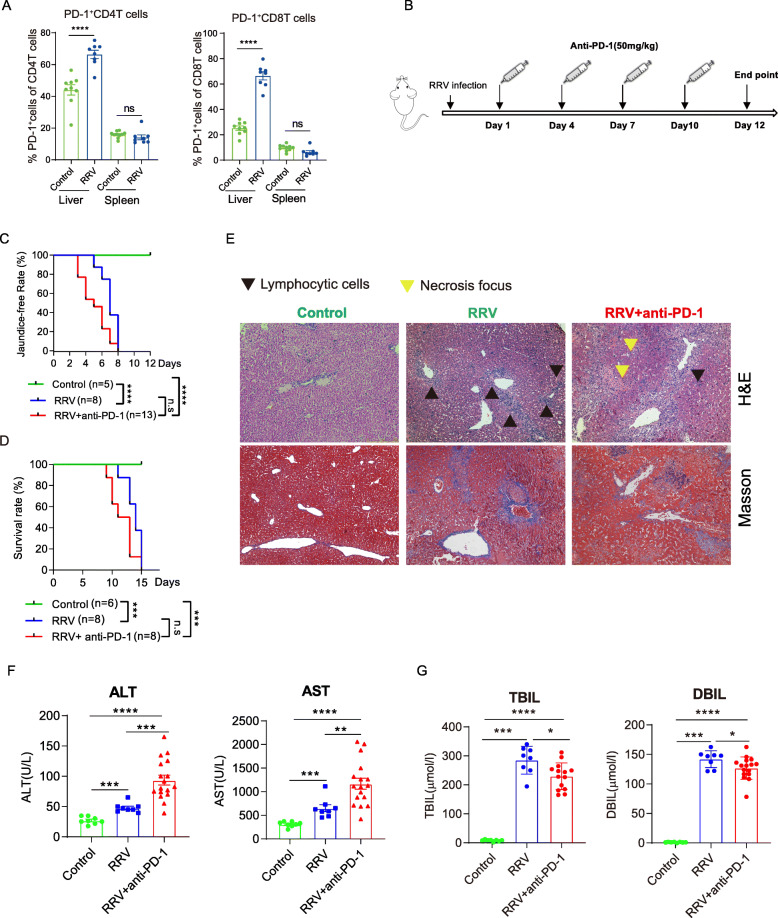
Blockade of PD-1 aggravates liver pathology in RRV-induced model of biliary atresia. **A** Frequency of PD-1^+^ cells in CD4^+^ and CD8 + T cells from livers and spleen of control (*n* = 9) and BA mice (*n* = 7). Data were expressed as mean ± SEM. Significance was determined by Mann-Whitney U test; ****p < 0.0001. **B** Schematic diagram of anti-PD-1 antibody administration. **C** Jaundice-free rates of control (*n* = 5), BA mice (*n* = 8), anti-PD-1 treated BA mice (*n* = 13). Significance was determined by Log-rank test. **D** Survival rate of control (*n* = 5), BA mice (*n* = 8), anti-PD-1 treated BA mice (*n* = 13). Significance was determined by Log-rank test. **E** Hematoxylin and eosin (H&E, × 100) staining showing liver section from control, BA mice, anti-PD-1 treated BA mice. Yellow and black tangles indicate necrosis focus and lymphocytic cells, respectively. **F** Dot plots showing concentration of ALT and AST of control (*n* = 5), BA mice (*n* = 8), and anti-PD-1 treated BA mice (*n* = 13). Data were expressed as mean ± SEM. Significance was determined by Mann-Whitney U test; ****p < 0.0001, ***p < 0.001, **p < 0.01, *p < 0.05. **G** Dot plots showing concentrations of total bilirubin (TBIL) and direct bilirubin (DBIL) of control (*n* = 5), BA mice (*n* = 8), and anti-PD-1 treated BA mice (*n* = 13). Data were expressed as mean ± SEM. Significance was determined by Mann-Whitney U test; ****p < 0.0001, ***p < 0.001,**p < 0.01, *p < 0.05

To validate these findings in vivo, we investigated an RRV-induced BA mouse model in neonatal BALB/c mice. We confirmed that PD-1 expression was significantly increased in CD4^+^ and CD8^+^ T cells in the liver of BA mice (Fig. 3A). We then administered an anti-PD-1 blocking antibody in RRV-infected mice (Fig. 3B). We found that blocking PD-1 tended to shift jaundice to an earlier time (Fig. 3C) and decrease overall survival rate compared with untreated RRV mice (Fig. 3D). Histologic analysis of livers also revealed necrosis foci and lymphocytes accumulation around the portal tracks in liver of both untreated and anti-PD-1 treated RRV mice (Fig. 3E). Furthermore, ALT and AST were significantly elevated (Fig. 3F) in anti-PD-1 treated RRV mice. Similar to findings in BA patients, we observed significant down-regulation of total and direct bilirubin concentrations in anti-PD-1 treated RRV-mice (Fig. 3G). These data evidenced that PD-1 expression play a protective role in limiting RRV-induced liver damage.

### Blockade of PD-1 augments IFN-γ expression in RRV-induced BA mice

**Fig. 4:**
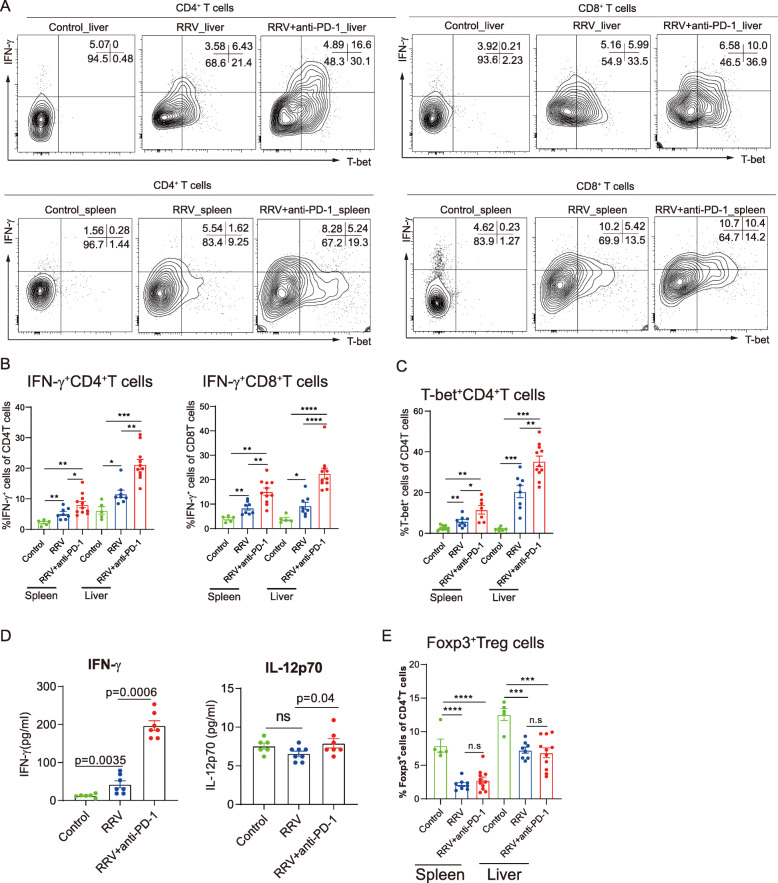
Blockade of PD-1 augments Th1 responses in RRV-induced BA mice. **A** Flow cytometry gating strategies for intrahepatic IFN-γ expressing and T-bet expressing CD4^+^T and CD8^+^T cells in liver and spleen from control, BA mice, anti-PD-1 treated BA mice. **B** Dot plots showing percentages of IFN-γ^+^CD8^+^T and IFN-γ^+^CD4^+^T cells in liver and spleen from control (*n* = 5), BA mice (*n* = 8), and anti-PD-1 treated BA mice (*n* = 13). Data were expressed as mean ± SEM. Significance was determined by Mann-Whitney U test, ****p < 0.0001,***p < 0.001,**p < 0.01. **C** Dot plots showing percentages of T-bet^+^CD4^+^ T cells in liver and spleen from control (*n* = 5), BA (*n* = 8), and anti-PD-1 treated BA mice (*n* = 13). Data were expressed as mean ± SEM. Significance was determined by Mann-Whitney U test,****p < 0.0001,***p < 0.001,**p < 0.01. **D** Dot plots showing plasma levels of IFN-γ and IL-12p70 in control (*n* = 5), BA mice (*n* = 8),and anti-PD-1 treated BA mice (*n* = 7). Data were expressed as mean ± SEM. Significance was determined by Mann-Whitney U test. **E** Dot plots showing percentage of Foxp3^+^Treg cells in liver and spleen from control (*n* = 5), BA mice (*n* = 8),and anti-PD-1 treated BA mice (*n* = 13). Data were expressed as mean ± SEM. Significance was determined by Mann-Whitney U test, ****p < 0.0001, ***p < 0.001, **p < 0.01

To confirm that PD-1 protects liver damage via suppressing IFN-γ expression in T cells, we next examined T cell function in RRV-induced BA model. We found that anti-PD-1 treatment promoted IFN-γ expression in both CD4^+^ and CD8^+^ T cells (Fig. 4A-B). T-bet, the master transcription factor of Th1, was also highly elevated in the liver of anti-PD-1 treated RRV mice (Fig. 4C). Consistently, plasma levels of IFN-γ and IL-12P70 were elevated by PD-1 blockade (Fig. 4D). Notably, anti-PD-1 treatment did not alter Foxp3^+^ Treg cells in RRV-induced BA model (Fig. 4E). Taken together, these data strengthen that PD-1 may have a protective role in alleviating BA liver damage by suppressing IFN-γ expression in T cells.

## Discussion

In the present study, we showed that PD-1 was highly elevated in CD4^+^ and CD8^+^ T cells in liver biopsies of infants with BA. High PD-1 expression was associated with low IFN-γ but higher bilirubin concentrations. Using RRV-induced BA model, we demonstrated that PD-1 directly suppressed IFN-γ but promoted bilirubin production.

PD-L1 is expressed on sinusoidal endothelial cells (LSEC), Kupffer cells, hepatocytes and stellate cells [18]. Interactions between PD-1^+^CD4^+^/CD8^+^T cells and PD-L1 expressing cells in liver of BA play a protective role in limiting BA pathogenesis. IFN-γ production was negatively correlated with the frequencies of PD-1^+^CD4^+^ and CD8^+^ T cells. PD-1 blockade in RRV induced BA mice contributed to an earlier onset of jaundice, enhanced Th1 response, and aggravated pathological injury of the liver. Unexpectedly, we found positive correlation between PD-1 expression and bilirubin concentration. When PD-1 was blocked, reduced bilirubin level was associated with abnormal increase of other liver function markers (ALT and AST). As a hormone, mildly elevated bilirubin levels have been newly established to be protective. Under inflammatory settings, bilirubin administration reduces TNF-α production by inhibiting iNOS but promoting prostaglandin E2 production [19].

## Conclusions

In summary, we found increased frequencies of PD-1 expressing CD4^+^ and CD8^+^T cells in the livers of infant with BA. We propose PD-1 elevation may limit liver damage by simultaneously suppressing IFN-γ production and oxidative damage. These findings may facilitate development of BA treatment strategies by harnessing the protective role of PD-1 signaling.

## Supplementary Information

**Additional file 1.**

**Additional file 2.**

## Data Availability

The datasets used and/or analyzed during the current study are available from the corresponding author on reasonable request.

## Abbreviations

BA: Biliary atresia
PD-1: Programmed death 1
RRV: Rhesus-rotavirus
T1D: Type 1 diabetes
SPF: Specific Pathogen Free
AST: Aspartate aminotransferase
GGT: γ-glutamyl transpeptidase
AKP: Alkaline phosphatase
ALT: Alanine aminotransferase
PBMC: Peripheral blood mononuclear cells
