# Supplementary material for: Programmed cell death protein-1 (PD-1) protects liver damage by suppressing IFN-γ expression in T cells in infants and neonatal mice
Source: BMC Pediatr. 2021 Jul 16;21:317. doi: 10.1186/s12887-021-02794-x (PMC8284022; doi:10.1186/s12887-021-02794-x)
Supplement: Supplementary file 1 — Additional file 1. [file 12887_2021_2794_MOESM1_ESM.docx]

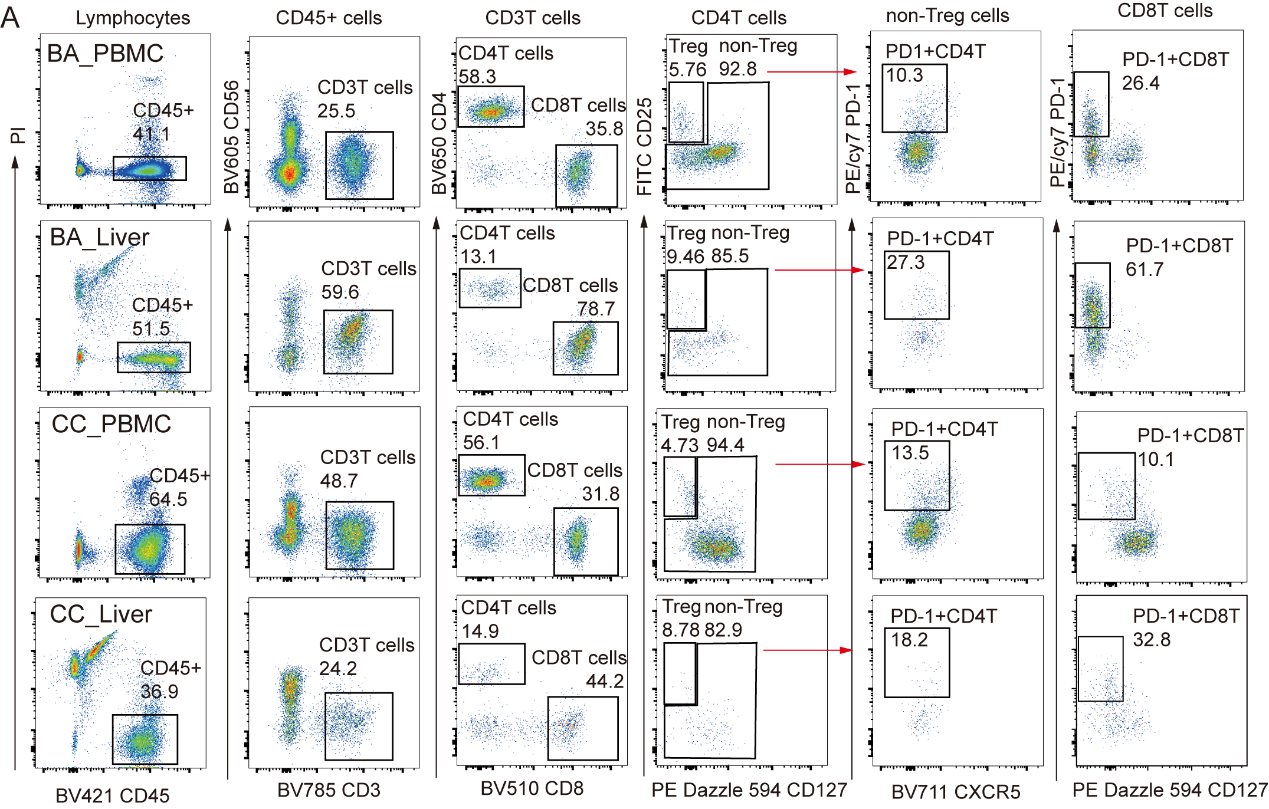


**Figure S1 Flow cytometry gating scheme of PD-1^+^CD4^+^ and CD8^+^ T cells in blood and liver of BA and CC subjects**

1. Flow cytometry gating scheme for intrahepatic CD3^+^ T, CD4^+^ T, CD8^+^ T, Treg, PD-1^+^CD4^+^T, PD-1^+^CD8^+^T for liver and PBMC from BA and CC infants.
