# Supplementary material for: Programmed cell death protein-1 (PD-1) protects liver damage by suppressing IFN-γ expression in T cells in infants and neonatal mice
Source: BMC Pediatr. 2021 Jul 16;21:317. doi: 10.1186/s12887-021-02794-x (PMC8284022; doi:10.1186/s12887-021-02794-x)
Supplement: Supplementary file 2 — Additional file 2. [file 12887_2021_2794_MOESM2_ESM.docx]

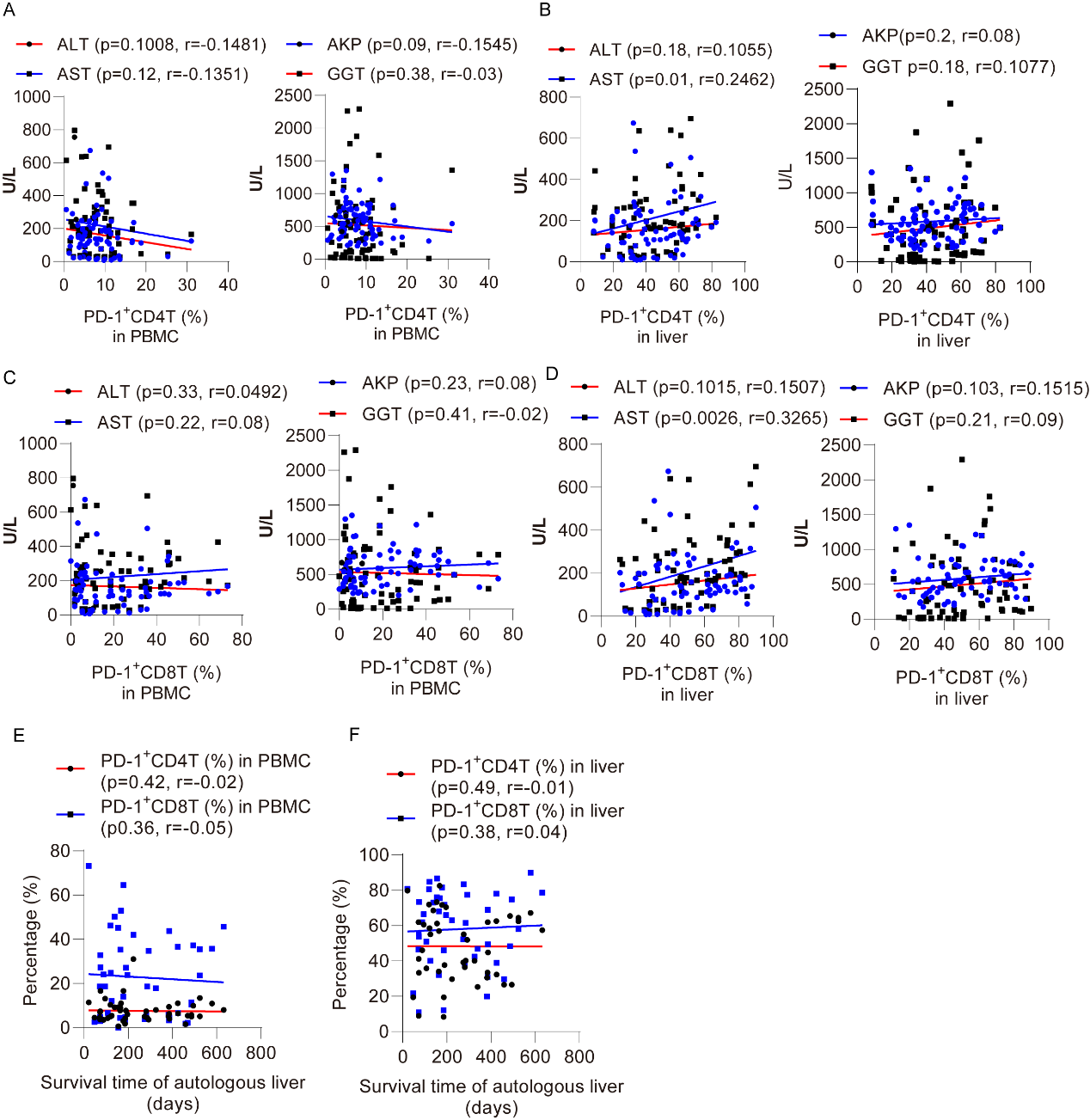


**FigureS2 Correlation between liver function indices and frequencies of PD-1^+^CD4^+^ and CD8^+^ T cells**

1. Scatter plots showing correlation between AST, ALT, AKP, GGT with the frequencies of PD-1^+^CD4^+^T cells in PBMC from BA and CC infants.
2. Scatter plots showing correlation between AST, ALT, AKP, GGT with the frequencies of PD-1^+^CD4^+^T cells in liver from BA and CC infants.
3. Scatter plots showing correlation between AST, ALT, AKP, GGT with the frequencies of PD-1^+^CD8^+^T cells in PBMC from BA and CC infants.
4. Scatter plots showing correlation between AST, ALT, AKP, GGT with the frequencies of PD-1^+^CD8^+^T cells in liver from BA and CC infants.
5. Scatter plots showing correlation between survival time of autologous liver with the frequencies of PD-1^+^CD4^+^ and CD8^+^ T cells in PBMC from BA patients.
6. Scatter plots showing correlation between survival time of autologous liver with the frequencies of PD-1^+^CD4^+^ and CD8^+^ T cells in liver from BA patients.
